# Tube current reduction and iterative image reconstruction for computed tomography myelography

**DOI:** 10.1038/s41598-024-79374-3

**Published:** 2024-11-12

**Authors:** Karolin J. Paprottka, Vivian Schultz, Karina Kupfer, Meinrad Beer, Claus Zimmer, Jan S. Kirschke, Thomas Baum, Nico Sollmann

**Affiliations:** 1 Department of Diagnostic and Interventional Neuroradiology, School of Medicine, Klinikum rechts der Isar, Technical University of Munich, Munich, Germany; 2 Department of Diagnostic and Interventional Radiology, University Hospital Ulm, Ulm, Germany; 3 TUM-Neuroimaging Center, Klinikum rechts der Isar, Technical University of Munich, Munich, Germany

**Keywords:** Dose reduction, Multi-detector computed tomography, Myelography, Image quality, Radiation exposure, Health care, Medical imaging, Medical research

## Abstract

This study aimed to systematically evaluate the impact of a low-dose (LD) protocol using tube current reduction on image quality, the confidence for intervention planning and guidance, and diagnostic yield for computed tomography (CT) myelography. We retrospectively analyzed 68 patients who underwent CT myelography, with 34 investigations performed with a standard-dose (SD) and 34 investigations performed with a LD protocol (using tube current reduction). The different scans were matched considering variables such as sex, age, presence of spinal instrumentation, and body diameter. All images were evaluated by two readers (R1 and R2) using Likert scales. Image noise was measured using attenuation values of paraspinal muscle tissue. Images were reconstructed with model-based iterative reconstruction (post-myelography diagnostic scans) or hybrid reconstruction (planning, periprocedural, and diagnostic scans). Image quality, overall artifacts, image contrast, and confidence for planning or intervention guidance were rated good to perfect for both SD and LD scans according to evaluations of both readers. Inter-reader agreement was good to very good for the images from intervention planning (κ ≥ 0.80) as well as for intervention guidance (κ ≥ 0.77), as well as for diagnostic scans (κ ≥ 0.85). Image noise was similar between SD and LD scans performed for planning of the interventional procedures (model-based iterative reconstruction: SD 45.37 ± 7.29 HU vs. LD 45.17 ± 9.12 HU; hybrid reconstruction: SD 46.05 ± 7.43 HU vs. LD 45.05 ± 8.69 HU; p > 0.05). The volume-weighted CT dose index (CTDI_vol_) and size-specific dose estimate (SSDE) were significantly lower for the planning scans as well as the periprocedural scans when using the LD protocol as compared to the SD protocol (p < 0.05). In conclusion, implementation of a LD protocol with tube current reduction for CT myelography is a feasible option to reduce radiation exposure, especially when combined with iterative image reconstruction. In our study, LD imaging did not have a relevant negative impact on image quality, confidence for intervention planning or guidance, or diagnostic certainty for CT myelography.

## Introduction

Myelography has played an important role as a diagnostic approach in the evaluation of spinal diseases for decades. Specifically, computed tomography (CT) myelography with prior intrathecal contrast agent administration represents a well-established diagnostic examination^1,2^. It combines the advantages of myelography with the high resolution of CT and thus enables the evaluation of different spinal pathologies that have contact to, displace, or impinge the thecal sac, the spinal cord, or the nerve roots^1,2^.

One of the most common indications for CT myelography is to evaluate the spinal canal and neural foramina in degenerative diseases, especially when the pathologic condition of concern is located in the intradural extramedullary space^1^. Compared to angiography-guided myelography, CT offers three-dimensional depiction of anatomy, and this may help in complicated cases in which plain angiography may not be sufficient to resolve complex morphological characteristics (e.g., in patients with severe scoliosis or degenerative changes). In such cases, the access route may be easier depicted by CT, and thus the procedure might be faster with CT. Furthermore, as compared to conventional magnetic resonance imaging (MRI) as another cross-sectional imaging method, CT delivers images with typically higher spatial resolution, hence CT myelography might be able to depict the location and configuration of the thecal sac in great detail, including visualization of even small nerve-related diseases as well as pathologies of the arachnoid space, such as arachnoid webs or adhesions or arachnoid cysts^2,3^. Furthermore, CT myelography can be a helpful tool for surgical planning as it enables an excellent depiction of osseous structures in combination with high-quality imaging of the thecal sac^1^. Moreover, CT myelography offers the possibility of dynamic/functional imaging of cerebrospinal fluid (CSF) spaces: the possibility of different patient positionings during the examination and the ability of obtaining delayed images makes the approach an excellent modality to evaluate abnormalities such as CSF leaks or fistulas that may be occult in single standard positions during scanning^2,4^. Furthermore, also in patients with spinal instrumentation, CT myelography remains still available for the investigation of spinal pathology^5^, especially in situations where MRI data would be non-diagnostic because of extensive hardware-related artifacts^6^. Last but not least, many other patient-related factors such as body habitus, claustrophobia, or inability to lay still for a long duration make CT myelography a useful alternative to MRI^1^.

However, despite its wide field of possible indications, CT myelography entails one major disadvantage as compared with MRI, which is the inevitable radiation burden^7^. Consequently, CT myelography is considered a rather high-dose imaging technique since its acquisition accounts for the major part of the collective effective dose for all radiographic examinations^8^. The volume-weighted CT dose index (CTDI_vol_), which indicates the average amount of radiation exposure emitted by the scanner, and on the other side, the dose-length product (DLP), which quantifies the total amount of ionizing radiation, are well established and known reference values to monitor radiation doses^9,10^. Both are provided by current state-of-the art scanners and can hence help to keep radiation exposure low^11^. Although dose reduction is important in clinical practice due to raising numbers of CT scans in the last years in clinical routine, there have only been few studies reporting on dose reduction in CT myelography and even less studies about dose reductions when discriminating between different parts of the spine^7,12^. Specifically, no studies with the combination of iterative image reconstruction and a matched-pair design are available regarding dose reduction in CT myelography, with a focus on image quality and confidence of the interventionalist for planning and performing the procedure.

Against this background, the aim of our study was to demonstrate that a low-dose (LD) protocol for CT myelography is a practical alternative to standard-dose (SD) approaches, maintaining overall image quality and confidence for the interventionalist for planning and performing a safe procedure at reduced radiation exposure to the patient.

## Material and methods

### Study cohort

This retrospective monocentric study with a matched-pair design was approved by the institutional review board of our Faculty of Medicine at the Technical University of Munich, and it was conducted in accordance with the Declaration of Helsinki. The requirement for written informed consent was waived due to this study’s retrospective design.

We retrospectively reviewed CT myelography procedures that had been performed with one of two different dose levels: SD or LD protocols for the planning and periprocedural scan (image reconstruction via iDose4; Philips Healthcare, Best, The Netherlands), as well as for the post-myelography diagnostic scan (image reconstruction with iDose4 and IMR1; Philips Healthcare, Best, The Netherlands). A general adjustment of our institutional CT protocols took place in October 2020, hence all LD scans included were acquired between November 2020 and April 2022, while all SD scans were derived from the interval between January 2020 and September 2020. The adjustment of scanning parameters was based on recent simulation studies from data of the herein used CT scanner regarding feasibility of LD imaging for the purpose of various clinical applications^13–15^. Prior to the distinct adjustment of our institutional CT protocols, the major reference settings for CT-based myelography procedures were as follows: tube potential of 120 kV (for planning and periprocedural scans) and 140 kV (for diagnostic post-myelography scans) and image reconstruction with IMR1 or iDose4. However, in clinical routine, deviations from the default tube potential were allowed based on best practice (considering factors such as patient habitus or presence of spinal implants), eventually requiring higher tube potentials for a respective scan in individual patients.

Patients were consecutively included if they had multi-detector CT (MDCT) scanning available in the context of CT-based myelography according to clinical indications. Patients were identified in the hospital’s institutional digital picture archiving and communication system (PACS). All eligible patient cases with LD scans were matched according to (1) sex (male/female), (2) age (± 15 years), (3) level of the post-myelography scan (holospinal, cervical, lumbar, thoracolumbar, or lumbosacral), (4) presence/absence of spinal instrumentation (metallic hardware causing artifacts in imaging data and making the access route to the target structure more demanding), and (5) body diameter (< 20 cm, 20–25 cm, 25–30 cm, and > 30 cm). Patients were excluded if (1) non-diagnostic image quality was present in MDCT data (e.g., due to motion artifacts), or (2) the planned intervention (including survey, planning, and periprocedural scans) was not accomplished (e.g., due to incompliance of the patient and preliminary abortion of the exam).

Overall, 68 cases were eligible and considered in this study (34 patients with SD scans and 34 matched patients with LD scans, 12 LD and 15 SD cases with only post-myelography diagnostic scans given that the puncture for intradural contrast agent administration was performed without image guidance).

### Multi-detector computed tomography

All scans included in this study were performed with the patient in prone position using one 128-slice MDCT scanner (Ingenuity Core 128; Philips Healthcare, Best, The Netherlands). If image guidance for the puncture was considered necessary on an individual case-by-case decision, after performing the scout scan covering the planned location of the puncture according to previous diagnostic imaging, a planning scan of the region to be considered was performed (spot scanning). The interventionalist then first selected the slice allowing for optimal visualization of the access route. During the subsequently performed interventional procedure when image guidance was considered, sequential scanning was achieved for guidance and surveillance during needle placement using a foot pedal (intermittent scanning, three axial images per shot). By default, images were reconstructed with model-based iterative reconstruction (post-myelography diagnostic scan: IMR1, Philips Healthcare, Best, The Netherlands) or hybrid reconstruction (planning, periprocedural, and post-myelography diagnostic scans: iDose4, Philips Healthcare, Best, The Netherlands). The parameters for the planning and periprocedural guidance scans as well as the diagnostic post-myelography scans are illustrated in Table 1, considering SD and LD protocols. For all SD and LD scans, a certain default windowing setting was provided, but during evaluation of the imaging data any windowing adjustments were allowed to improve image contrast.

**Table 1:** Details of the scanning protocol and image reconstruction for the planning and periprocedural guidance scans as well as the diagnostic post-myelography scans using a standard-dose (SD) or low-dose (LD) protocol for multi-detector computed tomography (MDCT).

	Planning scan		Periprocedural scan		Diagnostic post-myelography scan	
	SD	LD	SD	LD	SD	LD
Rotation time (in sec)	0.75		0.75		0.75	
Tube potential (range, in kV)	120–140		120		120–140	
Tube current (range, in mA)	40–333	20–67	60–200	30–60	248–485	182–409
Exposure (mean and range, in mAs)	120 kV: 46; 30–100 140 kV: 250	120 kV: 22; 15–50 140 kV: 32; 20–50	36; 30–100	17; 10–30	120 kV: 192; 22–307 140 kV: 345; 292–398	120 kV: 199; 103–289 140 kV: 131; 99–242
Collimation width (in mm)	0.625		0.625		0.625	
Image reconstruction	IMR1	IMR1	iDose4	iDose4	iDose4/IMR1	iDose4/IMR1
Reconstruction kernel	Bone and soft tissue	Bone and soft tissue	Bone and soft tissue	Bone and soft tissue	Bone and soft tissue	Bone and soft tissue

**Fig. 1:**
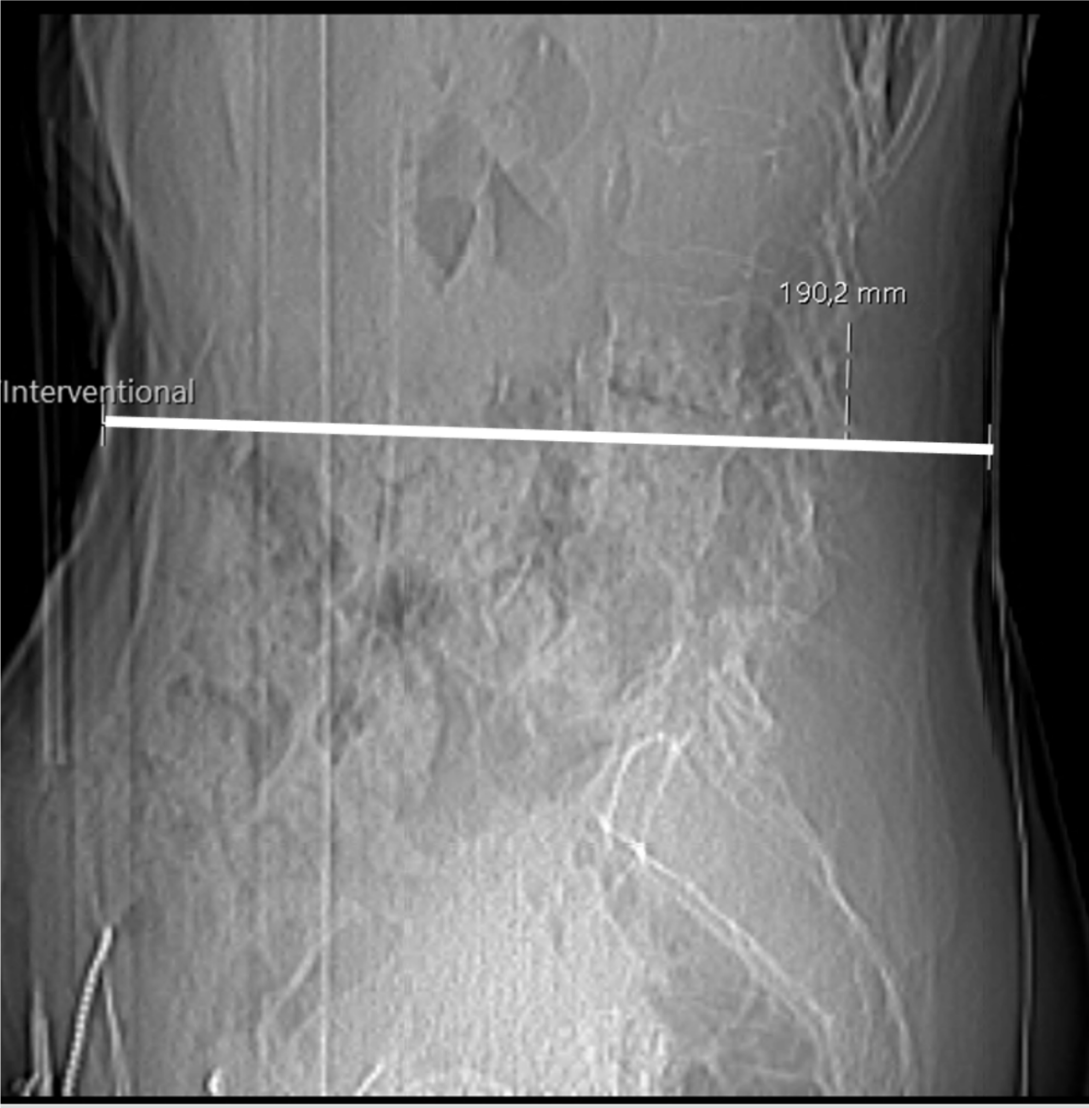
Lateral scout obtained for a planned computed tomography (CT) myelography in an 83-year-old female patient. The line indicates the anterior-posterior body diameter, which was equal to 190.2 mm in this exemplary case.

Parameters obtained from SD and LD scanning included the DLP, CTDI_vol_, scan parameters such as tube current and potential, and measurements of body diameter (in anterior–posterior direction). The individual body diameter was measured in the lateral scout scan (ideally at the level of the planned intervention) and was determined from skin-to-skin surface (Fig. 1)^16^. Based on the CTDI_vol_ and anterior–posterior body diameter, we systematically calculated the size-specific dose estimate (SSDE) for each patient and respective available scan (planning scan, periprocedural scan, and diagnostic post-myelography scan), which is given as the product of the CTDI_vol_ as displayed on the scanner and a body-diameter-related conversion factor as outlined by the American Association of Physicists in Medicine (AAPM)^17^. In this regard, CTDI_vol_ measures scanner output and not necessarily patient dose, hence previous work showed that the measured patient dose can be clearly different from the CTDI_vol_ as displayed by the scanner^9,18,19^.

### Image evaluation

**Fig. 2:**
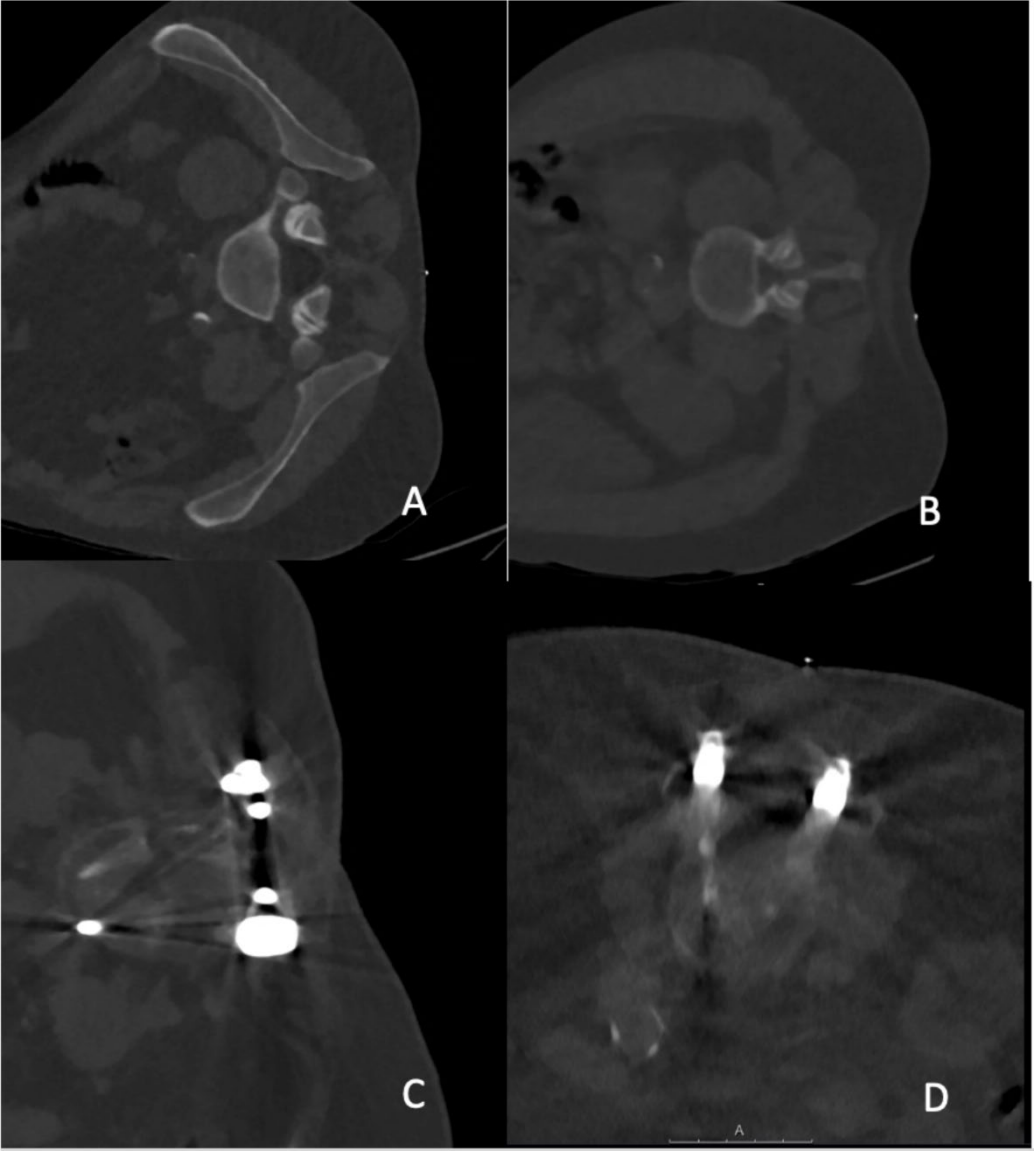
Examples for planning scans in different patients performed with a low-dose (LD) protocol for scanning, which were rated with perfect (**A**), good (**B**), medium (**C**), and poor (**D**) overall image quality*.* In cases (**C**) and (**D**), hardware of spinal instrumentation is partially covered by the imaging volume (bone kernel, windowing L = 750, W = 2500, slice thickness: 1 mm, IMR1).

After CT myelography, the imaging data were transferred to PACS, and for this study, the imaging data were evaluated with the standard PACS viewer (IDS7; Sectra AB, Linköping, Sweden) by two radiologists (board-certified radiologist with 10 years of experience, reader 1 [R1], and resident radiologist with 3 years of experience, reader 2 [R2]). The readers semi-quantitatively evaluated overall image quality, overall artifacts, image contrast, as well as confidence for intervention planning (based on planning scans), confidence for intervention guidance (based on the sequential scans during performance of the intervention), and diagnostic certainty, using 5-point or 3-point Likert scales (Table 2; Fig. 2).

**Table 2:** Semi-quantitative scoring for image evaluation by two readers.

Item	Score				
	1	2	3	4	5
Overall image quality	Very good to perfect	Good to very good	Medium	Poor	Very poor
Overall artifacts	None	Minimal	Prominent	Major	Severe
Image contrast	Very good to perfect	Good to very good	Medium	Poor	Very poor
Confidence for intervention planning (planning scans)	High	Medium	Low	x	x
Confidence for intervention guidance (sequential scans during the puncture)	High	Medium	Low	x	x
Diagnostic certainty	High	Medium	Low	x	x

During rating, both readers were strictly blinded to the rating results of each other. Furthermore, to minimize recall bias, a period of at least 3 weeks between the first and second rating of the data of different dose levels was adhered, and the images of the patients were presented in randomized order per each image reading round (SD or LD scans). Both readers were unaware of the distinct protocol used for scanning per reading round. The readers were presented with images using bone and soft tissue windowing, and they were allowed to manually adjust windowing levels if wanted, starting with a standard output in the PACS viewer (planning and guidance scan window level = 750, width = 2500; diagnostic scan window level = 1000, width = 900).

**Fig. 3:**
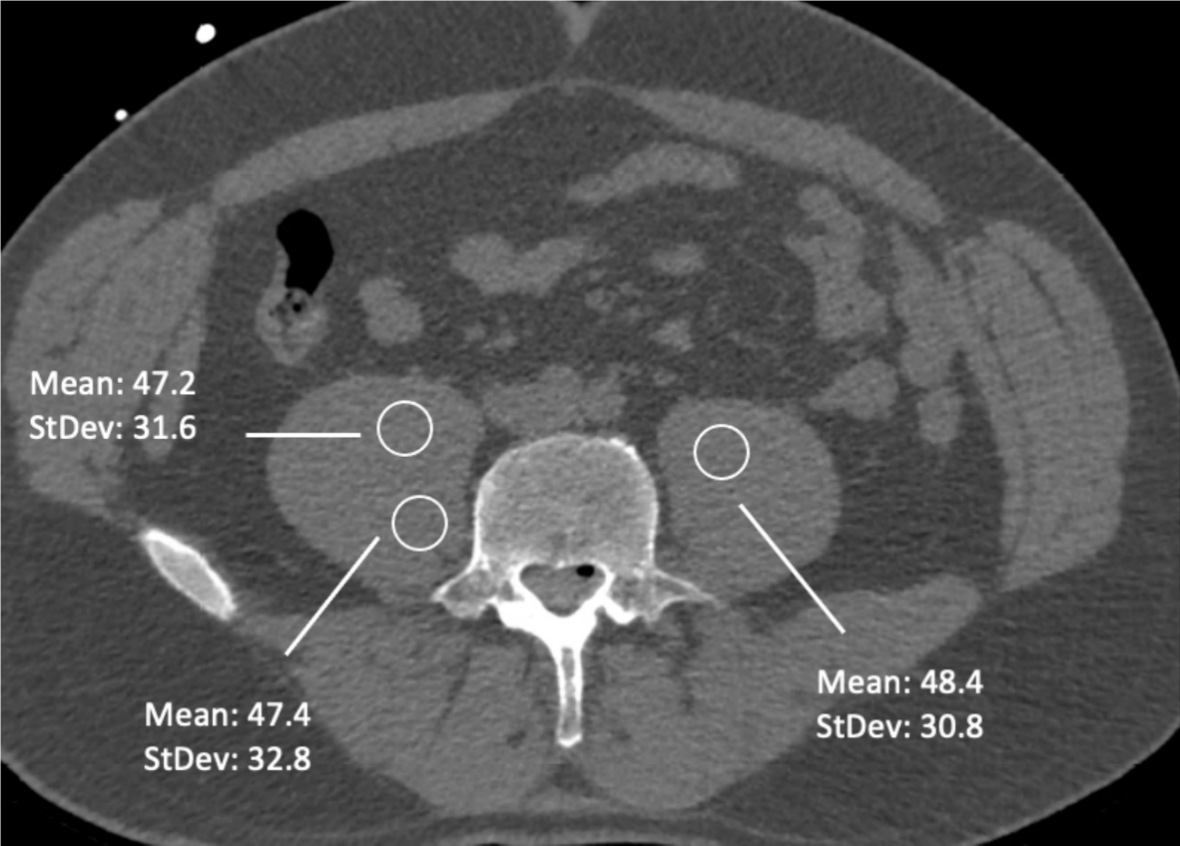
Quantitative estimation of image noise. Using three circular regions of interest (ROIs) in the psoas muscle per patient, the image noise was estimated by using the standard deviation (StDev) of the attenuation in Hounsfield units (HU).

In addition to the semi-quantitative rating described above with Likert scales, a quantitative evaluation was performed. Thereby image noise (ideally at the level of the procedure) was estimated by manually placing a ~ 10-mm^2^ circular region of interest (ROI) in the psoas muscles in the diagnostic post-myelography scans, and the standard deviation (StDev) of the attenuation in Hounsfield units (HU) of the psoas muscles was then documented^16,20^. For each included patient case, three separate measurements were performed (Fig. 3) by one reader (R1). Thereafter, these three obtained values from the ROIs were averaged per patient.

### Statistical analysis

For statistical data analysis, SPSS software (version 29.0; IBM SPSS Statistics for Windows, IBM Corp., Armonk, NY, USA) and Prism software (version 10.3.1; GraphPad Software, Boston, MA, USA) were used, and a p-value < 0.05 (two-sided) was set as the threshold for statistical significance for all tests. For sensitivity analysis, G*Power software (version 3.1.9.7; https://www.psychologie.hhu.de/arbeitsgruppen/allgemeine-psychologie-und-arbeitspsychologie/gpower) was used.

Descriptive statistics were calculated for the scores derived from evaluations of each reader as well as for the attenuation measurements from ROIs. Furthermore, descriptive statistics were calculated for patient demographics and dose measurements (CTDI_vol_, DLP, and SSDE). Inter-reader agreement for scorings of both readers concerning overall image quality, overall artifacts, image contrast, and confidence or diagnostic certainty was evaluated with weighted Cohen’s kappa (κ). To compare the scores between SD and LD scans, Wilcoxon signed-rank tests were performed for the evaluations of each reader, respectively. Furthermore, Wilcoxon signed-rank tests were also conducted to compare demographics between groups.

For dose characteristics including DLP, CTDI_vol_, and SSDE for the available planning scans, periprocedural scans, and diagnostic post-myelography scans, single Wilcoxon signed-rank tests were performed to assess differences between acquisitions with the SD versus LD protocols. Similarly, for image noise, also Wilcoxon signed-rank tests were performed for HU values. Those tests were followed up by a mixed-effects analysis for the dose data and image noise data, respectively, with Bonferroni multiple comparisons test as a post-hoc assessment. For the dose data, a post-hoc sensitivity analysis was added (alpha error probability of 0.05, power of 0.80).

## Results

### Patient cohort

**Fig. 4:**
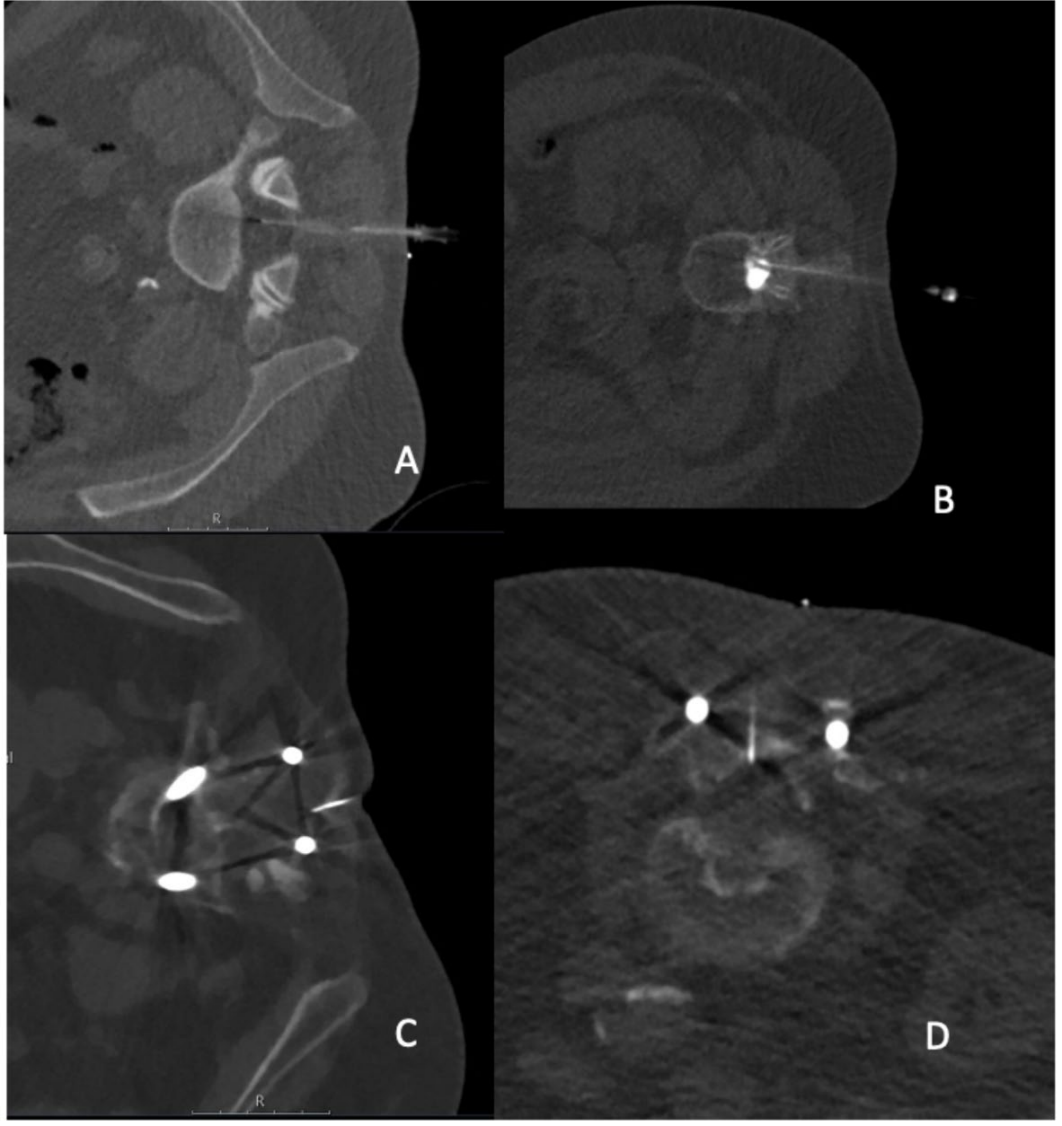
Examples for image-guided punctures at the lumbar spine for administration of intradural contrast agent prior to computed tomography (CT) myelography in different patients performed with a low-dose (LD) protocol for scanning, with resulting CT scans rated as perfect (**A**; 120 kV; 30 mA; CTDI_vol_ 1.2 mGy × 12; DLP 14.4 mGy*cm), good (**B**; 120 kV; 30 mA; CTDI_vol_ 1.2 mGy × 1; DLP 1.2 mGy*cm), medium (**C**; 140 kV; 27 mA; CTDI_vol_ 1.2 mGy × 1; DLP 1.2 mGy*cm), and poor (**D**; 120 kV; 30 mA; CTDI_vol_ 2.4 mGy × 9; DLP 21.6 mGy*cm) regarding overall image quality (**A**–**D**: bone kernel; windowing L = 750, W = 2500, slice thickness: 3.33 mm, iDose4).

**Fig. 5:**
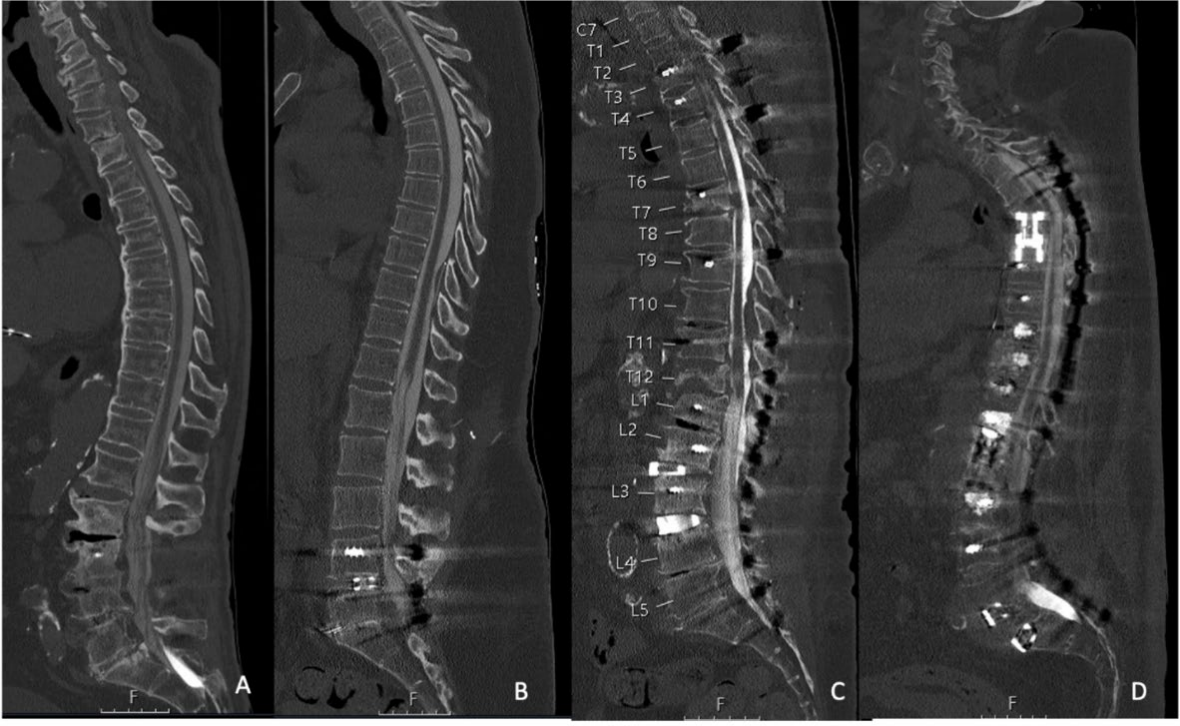
Examples for computed tomography (CT) myelography scans (after intradural contrast agent administration) in different patients performed with a standard-dose (SD) protocol for scanning, which were rated with perfect (**A**; L = 750, W = 2500, 140 kV, 319 mA, bone kernel; 2 mm, iDose4, CTDI_vol_ 13.4 mGy), good (**B**; L = 750, W = 2500, 120 kV, 494 mA, bone kernel; 2 mm, iDose4, CTDI_vol_ 19.7 mGy), medium (**C**; L = 750, W = 2500, 120 kV, 347 mA, bone kernel; 2 mm, iDose4, CTDI_vol_ 18.2 mGy), and poor (**D**; L = 750, W = 2500, 140 kV, 418 mA, bone kernel; 2 mm, iDose4, CTDI_vol_ 38.8 mGy) overall image quality.

**Fig. 6:**
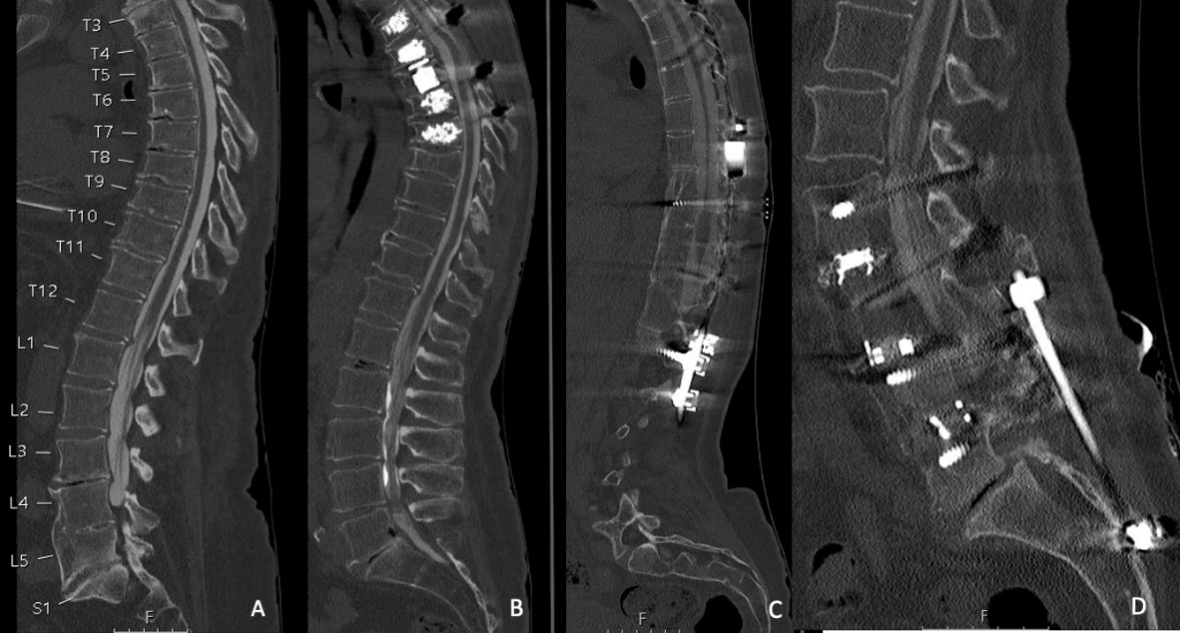
Examples for computed tomography (CT) myelography scans (after intradural contrast agent administration) in different patients performed with a low-dose (LD) protocol for scanning, which were rated with perfect (**A**; L = 750, W = 2500, 140 kV, 287 mA, bone kernel; 2 mm, iDose4, CTDI_vol_ 13.6 mGy), good (**B**; L = 750, W = 2500, 140 kV, 294 mA, bone kernel; 2 mm, iDose4, CTDI_vol_ 9.8 mGy), medium (**C**; L = 750, W = 2500, 140 kV, 281 mA, bone kernel; 2 mm, iDose4, CTDI_vol_ 15.4 mGy), and poor (**D**; L = 750, W = 2500, 140 kV, 308 mA, bone kernel; 2 mm, iDose4, CTDI_vol_ 10.3 mGy) overall image quality.

A total of 68 patients were enrolled, with 34 patients assigned to the SD imaging group and the other 34 patients assigned to the LD imaging group. According to matching criteria, in both groups 12 patients were female and 22 patients were male (Table 3). For each patient, the diagnostic post-myelography scan and if available the planning and periprocedural scans were rated (not all punctures were done under CT surveillance: 22 patients scanned with LD and 19 patients scanned with SD protocols underwent myelography with CT-guided punctures). In most cases, the thoracolumbar spine was covered by the diagnostic post-myelography scans. Per group, 23 patients showed implants from previous surgery within the field of view at the spine. During the intervention, no major complications (e.g., bleeding) were reported for any of the myelography procedures performed either with the SD or LD protocol. Figures 4, 5 and 6 depict exemplary patient cases.

**Table 3:** Overview of patient characteristics, dose parameters including volume-weighted computed tomography dose index (CTDI_vol_), dose-length product (DLP), and size-specific dose estimate (SSDE), and image noise measured in Hounsfield units (HU).

		SD	LD	P-value
Sex		12 females, 22 males	12 females, 22 males	Matching criteria (p > 0.05)
Age (mean and range, in years)		69; 29–89	67; 28–93	
Body diameter (anterior–posterior; mean and range, in mm)		286; 201–462	283; 201–445	
Dose planning scan (mean and StDev, in mGy resp. mGy*cm)	CTDI_vol_	5.06 ± 6.32	1.84 ± 1.09	*0.004*
	DLP	48.70 ± 86.09	16.17 ± 7.78	*0.01*
	SSDE	2.33 ± 2.50	0.94 ± 0.55	*0.007*
Dose periprocedural scan (mean and StDev, in mGy resp. mGy*cm)	CTDI_vol_	2.86 ± 1.34	1.43 ± 0.56	< *0.001*
	DLP	30.57 ± 30.63	11.43 ± 9.49	0.06
	SSDE	1.42 ± 0.42	0.76 ± 0.38	< *0.001*
Dose diagnostic post-myelography scan (mean and StDev, in mGy resp. mGy*cm)	CTDI_vol_	14.39 ± 6.18	12.76 ± 4.02	0.48
	DLP	594.86 ± 367.76	541.81 ± 324.42	0.95
	SSDE	7.31 ± 2.09	6.64 ± 1.36	0.34
Image noise diagnostic post-myelography scan (mean and StDev, in HU)	iDose4	46.05 ± 7.43	45.05 ± 8.69	0.71
	IMR1	45.37 ± 7.29	45.17 ± 9.12	0.83

Values are provided as means ± standard deviation (StDev) and/or ranges for imaging performed with standard-dose (SD) or low-dose (LD) protocols. P-values indicating a statistically significant difference (p < 0.05) are highlighted by italic font.

### Semi-quantitative evaluation

Image quality, overall artifacts, and image contrast in all scans as well as confidence for planning (planning scan), intervention guidance (scan during intervention), as well as the diagnostic certainty in the diagnostic scans were rated good to perfect on average for both SD and LD scans according to evaluations of both readers, with only statistically significant differences between SD vs. LD scans for confidence for intervention planning for both readers with p = 0.02 and p = 0.01, respectively (Tables 4, 5, 6, 7). For all other parameters, no statistically significant differences were detected (p > 0.05). Further, inter-reader agreement was good to very good for the images from intervention planning (range of κ: 0.80–1.00) as well as from intervention guidance (range of κ: 0.77–0.93), and the diagnostic scans for either iDose4 (range κ: 0.85–0.96) or IMR1 (range of κ: 0.85–0.98; Tables 4, 5, 6, 7).

**Table 4:** Semi-quantitative scoring for intervention planning scans according to evaluations of two readers (R1 and R2) considering scanning with standard dose (SD) and low dose (LD).

Intervention Planning			
	LD	SD	P-value
Overall Image Quality			
R1	1 (1–4)	1 (1–4)	0.57
R2	1 (1–3)	1 (1–4)	0.87
Kappa	0.80	0.85	
Overall Artifacts			
R1	1 (1–4)	1 (1–4)	0.13
R2	1 (1–4)	1 (1–4)	0.06
Kappa	0.94	0.94	
Image Contrast			
R1	1 (1–4)	1 (1–4)	0.44
R2	1 (1–3)	1 (1–4)	0.35
Kappa	0.93	1.00	
Confidence for Intervention Planning			
R1	1 (1–3)	1 (1–3)	*0.02*
R2	1 (1–2)	1 (1–3)	*0.01*
Kappa	0.80	0.96	

P-values indicating a statistically significant difference (p < 0.05) are highlighted by italic font.

**Table 5:** Semi-quantitative scoring for periprocedural guidance scans according to evaluations of two readers (R1 and R2) considering scanning with standard dose (SD) and low dose (LD).

Intervention Guidance			
	LD	SD	P-value
Overall Image Quality			
R1	1 (1–3)	1 (1–3)	0.66
R2	1 (1–3)	1 (1–3)	0.05
Kappa	0.89	0.92	
Overall Artifacts			
R1	1 (1–3)	1 (1–4)	0.34
R2	1 (1–3)	1 (1–4)	0.43
Kappa	0.85	0.93	
Image Contrast			
R1	1 (1–3)	1 (1–4)	0.13
R2	1 (1–2)	1 (1–3)	0.26
Kappa	0.86	0.90	
Confidence for Intervention Guidance			
R1	1 (1–2)	1 (1–3)	0.41
R2	1 (1–2)	1 (1–3)	0.32
Kappa	0.77	0.86

**Table 6:** Semi-quantitative scoring for the diagnostic post-myelography scans with hybrid image reconstruction (via iDose4) according to evaluations of two readers (R1 and R2) considering scanning with standard dose (SD) and low dose (LD).

Diagnostic Scan (iDose4)			
	LD	SD	P-value
Overall Image Quality			
R1	1 (1–4)	1 (1–4)	0.86
R2	1 (1–3)	1 (1–3)	0.66
Kappa	0.93	0.89	
Overall Artifacts			
R1	1 (1–4)	1 (1–4)	0.60
R2	1 (1–3)	1 (1–4)	0.49
Kappa	0.96	0.96	
Image Contrast			
R1	1 (1–3)	1 (1–4)	0.84
R2	1 (1–2)	1 (1–3)	0.83
Kappa	0.92	0.88	
Diagnostic Certainty			
R1	1 (1–3)	1 (1–3)	1.0
R2	1 (1–2)	1 (1–3)	1.0
Kappa	0.85	0.88

**Table 7:** Semi-quantitative scoring for the diagnostic post-myelography scans with model-based iterative image reconstruction (via IMR1) according to evaluations of two readers (R1 and R2) considering scanning with standard dose (SD) and low dose (LD).

Diagnostic Scan (IMR1)			
	LD	SD	P-value
Overall Image Quality			
R1	1 (1–4)	1 (1–4)	0.24
R2	1 (1–4)	1 (1–3)	0.27
Kappa	0.90	0.94	
Overall Artifacts			
R1	1 (1–4)	1 (1–4)	0.85
R2	1 (1–3)	1 (1–4)	0.80
Kappa	0.95	0.98	
Image Contrast			
R1	1 (1–3)	1 (1–4)	0.58
R2	1 (1–3)	1 (1–3)	0.67
Kappa	0.92	0.90	
Diagnostic Certainty			
R1	1 (1–3)	1 (1–3)	0.83
R2	1 (1–2)	1 (1–3)	0.81
Kappa	0.85	0.87	

### Radiation exposure

The DLP as well as the CTDI_vol_ and SSDE were significantly lower for the planning scans when comparing the SD to the LD protocols (p < 0.05 each; Table 3). For the periprocedural scans, both the CTDI_vol_ and SSDE ware significantly lower for the LD protocol (p < 0.05 each; Table 3). Regarding the DLP, CTDIvol, and SSDE for the diagnostic post-myelography scans, differences between protocols were not statistically significant (p > 0.05, Table 3).

Regarding the results of the mixed-effects analysis, an overall significant difference was observed for CTDI_vol_ (p < 0.01; F(1.296, 28.77) = 60.75; Bonferroni multiple comparisons test statistically significant for guidance scans with adjusted p-value of 0.01) as well as for SSDE (p < 0.01; F(1.437, 31.90) = 116.9; Bonferroni multiple comparisons test statistically significant for planning scans with adjusted p-value of < 0.01) and DLP (p < 0.01; F(2.065, 46.68) = 45.45; Bonferroni multiple comparisons test without statistically significant results). The related effect size for post-hoc sensitivity analysis indicated a small effect size.

### Image noise

Noise according to quantitative evaluation using muscle attenuation values was similar between SD and LD scans for IMR1 and iDose4 reconstructions, as measured in the diagnostic post-myelography scans (SD IMR: 45.37 ± 7.29 HU vs. LD IMR: 45.17 ± 9.12 HU, p > 0.05; SD iDose4: 46.05 ± 7.43 HU vs. LD iDose4: 45.05 ± 8.69, p > 0.05; Table 3). Regarding the results of the mixed-effects analysis, no overall significant difference was observed for image noise (p = 0.64; F(1.331, 61.23) = 0.3147; Bonferroni multiple comparisons test without statistically significant results).

## Discussion

Our study analyzed the impact of dose reduction by lowered tube currents on image quality, the confidence for intervention planning, guidance, and the diagnostic scans of CT myelography. We were able to show that dose reduction for planning and periprocedural guidance scans as well as the diagnostic scans of myelography with MDCT is feasible and can be performed without clinically relevant drawbacks regarding image quality or confidence. The DLP as well as the CTDI_vol_ and SSDE were significantly lower for the planning scans when using the LD protocol as compared to the SD protocol, and the CTDI_vol_ and SSDE were significantly lower for the periprocedural scans as well. We did not find a statistically significant difference between the LD and SD protocol for diagnostic post-myelography scans regarding CTDI_vol_, SSDE, or DLP in individual comparisons; however, in the overall mixed-effects analysis, a significant difference was obtained though. Image quality, overall artifacts, and image contrast in all scans as well as confidence for planning (planning scan), intervention guidance (scan during intervention), as well as the diagnostic certainty in the diagnostic post-myelography scans were rated good to perfect for both SD and LD scans according to evaluations of both readers. Overall, noise according to quantitative evaluation using muscle attenuation values was similar between the SD and LD protocols.

Image-based guidance for myelography is a commonly used procedure in patients with findings in need for clarification^12,21^. Assessing and monitoring dose data of CT examinations helps to ensure radiation protection and optimize CT protocols^22^. Yet, concerns with CT-based guidance relate to potential consequences of ionizing radiation. Hence, there are many efforts to keep radiation exposure as low as reasonably achievable (ALARA principle)^23–25^. The options available for reaching the goal of low radiation doses in CT are manifold and primarily include adaptions in scanning parameters such as for tube current, tube potential, slice thickness, patient coverage, number of acquisitions, and/or length of the procedure^23^. In the course of CT scanning, protocol optimizations, and routine introduction of model-based iterative reconstruction, we adjusted the CT protocol based on a former conventional SD protocol to provide LD scanning with reduced radiation exposure. Previously published in-vivo studies demonstrated the utility of LD techniques for a multitude of interventional procedures^20,26^. Yet, among these studies only few studies exist that are dealing with dose aspects for the diagnostic scan of CT myelography, such as the investigation performed by Nicholson et al.^12^, who compared radiation dose parameters for CT myelograms and digital-subtraction myelograms for patients with possible CSF leaks. For CT myelography, they reported a median CTDI_vol_ of about 38 mGy (range 10–104 mGy) and a DLP of about 1185 mGy*cm (range 186–4848 mGy*cm) for imaging of the whole spine^12^. Thielen et al. reported on dose data in a series of 14 patients with high-flow CSF leaks caused by spinal osteophytes, who underwent ultra-fast dynamic CT myelography^27^. They reported a CTDI_vol_ of 21.4 mGy and a mean effective dose of 70.6 mSv (range 21.5–182.9 mSv)^27^. Furthermore, the research group of Dobrocky et al. also reported on dose data in dynamic CT myelography scans in patients with CSF leaks^28^. They described a mean CTDI_vol_ of 107 mGy (range 12–246 mGy) and DLP of 1347 mGy*cm (range 550–3750 mGy*cm)^28^. Zensen et al. compared radiation doses of single- and dual-source examinations in a retrospective study (183 CT myelographies comprising 155 single-source and 28 dual-source examinations; automatic tube current modulation with an exposure control system)^7^. Dose data included 31 whole-spine, 119 lumbar, 10 thoracic, and 23 cervical CT myelography exams, with a median CTDI_vol_ of 7.44 mGy (range 4.25–16.15 mGy) and DLP of 509.7 mGy*cm (279.6–1033.0 mGy*cm) for the whole-spine exams^7^.

In comparison with these studies^7,12,27,28^, our determined radiation exposures were similarly low or even lower, yet we performed a dedicated analysis of the planning scans, periprocedural scans, as well as diagnostic post-myelography scans in the present work. Furthermore, we used a standard MDCT scanner for the procedures combined with hybrid or model-based iterative image reconstruction, hence our results may be transferrable to other institutions using widely available CT hardware and reconstruction approaches that are increasingly available for the clinical routine. The decreased radiation exposure in our study can be explained by modified protocol settings. Recent techniques of dose reduction have also focused on iterative image reconstruction models such as adaptive statistical iterative reconstruction or model-based iterative reconstruction^13–15^. Therefore, in our study, the planning scans (iDose4), the interventional guidance scans (iDose4), and the diagnostic scans (iDose4 and IMR1) were reconstructed using hybrid or model-based iterative reconstruction algorithms, given that especially model-based iterative approaches may help to increase the visibility of anatomical details whilst facilitating LD protocols^29–31^. In the future, a wider availability could lead to an increased acceptance of model-based iterative reconstruction algorithms for the radiologist while evaluating images with LD regimens during CT-guided interventions and, therefore, potentially result in further reductions of the radiation dose applied to both the interventional radiologist as well as the patient.

Lowering the tube current for MDCT can be a simple and effective method for reducing radiation exposure to both the patient and interventionalist regarding the diagnostic scans of the CT myelography as well as the prior CT-guided puncture, if done with an image-guided approach. In addition to the post-contrast images after the procedure, CT-guided spinal interventions usually comprise different phases, including survey images, planning images, as well as the guidance images during the procedure, which all contribute to the overall radiation exposure. Thus, there is an opportunity to reduce doses in all of these steps. A review by Sarti et al. showed that as much as approximately 89% of the total radiation exposure during CT-guided interventions such as biopsy, drainage, and ablation was caused by the planning scans^32^. In our study, we investigated the effect of dose reduction in all consecutive parts of the CT-based procedures. Our results may indicate that LD imaging with adjustments of the tube current can lead to a significant reduction of the DLP and CTDI_vol_ in at least the planning and periprocedural scans–without any relevant loss of image quality or diagnostic certainty. Although we matched patients, differences of patients’ characteristics between SD and LD groups, such as precise diameters and influences on the field of view or the size/length of the metal device, could also influence radiation doses especially in the diagnostic scan. Our results are in line with results from former work where the impact of dose reduction in CT-guided spine biopsies^26^ and periradicular therapies were investigated^20^. In these studies, the DLP and CTDI_vol_ were also lower for LD scans regarding the planning scans as well as the periprocedural guidance scans while image quality, image contrast, the determination of the target structure, and confidence for planning or intervention guidance were rated good to perfect for both SD and LD scans without statistically significant differences between SD versus LD scans^20,26^. In correlation to the results of our current study, these former studies also found that a reduction of the radiation dose via tube current lowering can entail a significant reduction of the DLP regarding the planning scans as well as the interventional guidance scans (without relevant drawbacks regarding image quality or confidence)^20,26^. From this point of view, pre-myelography imaging should be carefully reviewed and when possible focus on the definite area of interest, together with an optimized protocol selection to obtain images from scans with low radiation doses.

Yet, there are some limitations to this study. First, the study design was retrospective and performed at a single academic medical center, implicating that CT myelography was conducted by different interventionalists with different education levels. Therefore, the reproducibility of the results using the new LD protocol cannot be fully assessed in this study. Second, the lack of quantifying artifacts associated with the metallic needle or metallic implants can be considered a limitation, which can lead to restrictions in overall image quality and thus can have impact on periprocedural guidance. Moreover, the presence of spinal hardware may entail increased radiation doses to obtain images with sufficient quality when using automatic tube current modulation, thus potentially relating to DLP and CTDI_vol_ values that were not statistically significantly different between the LD and SD examinations for diagnostic post-myelography imaging. Besides, we have to acknowledge that the LD protocol may not be suitable for all patients or clinical scenarios. For example, in patients with severe spinal stenosis or extensive spinal instrumentation, the LD protocol may not provide adequate image quality for diagnostic purposes or dedicated intervention planning. Furthermore, due to the small size of the patient cohort, it is recommended to investigate radiation exposure in a larger population in multicenter studies, which could be the next necessary step for setting general reference levels. Related to this, we also have to acknowledge that data of this study stem from a single-center study using imaging data from one scanner and one SD and LD protocol, respectively, which limits the generalizability of findings. Future studies are required for investigations of different scanners and other potentially promising LD protocols.

## Conclusion

Considerable dose reductions for CT myelography can be realized by tube current lowering and hybrid or model-based iterative image reconstruction, in particular for planning scans as well as periprocedural scans. In our present study, application of the LD protocol did not significantly impact image quality or diagnostic certainty.

## Data Availability

The datasets used and analyzed during the current study are available anonymized from the corresponding author on reasonable request.
